# Erratum

**DOI:** 10.1155/MBD.1995.241

**Published:** 1995

**Authors:** R. C. Elder


					ERRATUM
R.C. Elder, W.B. Jones Z. Zhao, J.G. Dorsey, K. Tepperman,
Myochrysine Solution Structure and Reactivity,
Metal-Based Drugs 1994, 1,363.
Equation (2) on page 369 should read:
[TMAuCN] + [CN]
K2
[Au(CN)2] + [TM] (2)
Equation (3) on page 369 should read:
Kapp KI*K2 {[Au(CN)2]-, [TM]'}/{[AuTM]. [(CN)-]2} 600.0 M1 (3)
Most importantly the value of Kapp is 600.0 M
-not 0.0006 M
-as originally printed.
thank Professor C. Frank Shaw, III, for bringing the error in the numerical value of Kapp to my
attention.
Richard C. Elder
241

				

